# Model Membrane‐Free Li–S Batteries for Enhanced Performance and Cycle Life

**DOI:** 10.1002/advs.201500068

**Published:** 2015-04-15

**Authors:** Kenville E. Hendrickson, Lin Ma, Gil Cohn, Yingying Lu, Lynden A. Archer

**Affiliations:** ^1^ School of Chemical and Biomolecular EngineeringCornell University120 Olin HallIthacaNY14853USA; ^2^ Department of Materials Science and EngineeringCornell UniversityIthacaNY14853USA

**Keywords:** dissolved polysulfide, high‐energy batteries, membrane‐free batteries, sulfur batteries

## Abstract

**The success of the rechargeable Li–S cell** is limited in part by the dissolution of lithium‐polysulfide in the electrolyte. Remarkably, it is found that removal of the conventional membrane separator in a Li–S cell improves sulfur utilization and cycling performance, whether the sulfur is initially contained in the cathode or electrolyte. An optimized cell design yields discharge capacities as high as 980 mA h g^−1^ after 100 cycles.

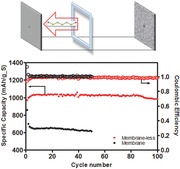

Advanced energy storage systems are increasingly reliant on rechargeable lithium ion batteries (LIBs). The portability, low rates of self‐discharge, high voltage, and high volumetric energy density relative to other rechargeable battery platforms make LIBs attractive in multiple economic sectors, including transportation. With LIBs performance now approaching theoretical limits,^1^ there is growing concern that this workhorse storage technology will not meet emerging energy storage demands for transportation and for autonomous vehicles. Secondary batteries with significantly higher energy densities compared to current LIBs are required for meeting these needs. With a high theoretical capacity of 1675 mA h g^−1^, sulfur is fast becoming one of the most studied cathode materials for such high‐energy cells.^2^ Sulfur achieves its high energy by hosting two lithium ions non‐topotactically in a multistep electrochemical redox reaction that can be summarized as follows: 16Li + S_8_ → 8Li_2_S.^3^, ^4^

Several recent publications,^4^, ^5^, ^6^, ^7^, ^8^ including two excellent reviews,^9^, ^10^ summarize the promise of Li–S cells as rechargeable storage platforms and outline many of the key barriers that must be overcome for the technology to live up to the promise set by its chemistry. Among the most studied barriers, loss of active material from the cathode to the electrolyte has received greatest attention because it results in poor cathode utilization, self‐discharge, and short cycle life.^11^, ^12^, ^13^ This is attributed to dissolution of lithium polysulfide cathode intermediates (Li_2_S*_x_*, 2 < *x* < 8) to the electrolyte. Additional barriers stemming from poor electrical conductivity of sulfur and its reduction products in the cathode have also received significant attention.^14^ The loss of Li_2_S*_x_* to the electrolyte during discharge is arguably the more challenging of these barriers because dissolution of Li_2_S*_x_* appears necessary to some extent for proper cell functioning. In particular, dissolved lithium poly­sulfide intermediates (Li‐PS) can help lower the high charge transfer resistance of the cathode caused by the poor electrical conductivity of sulfur and its reduction products, by concentrating redox events at the electrolyte/carbon interfaces in the cathode. Li‐PS dissolution is now understood to begin with the formation of liquid high order Li‐PS (Li_2_S*_x_*, 4 ≤ *x* ≤ 8) in a redox process that occurs between 2.4 and 2.0 V. Further electrochemical reduction below 2.0 V yields low order PS (Li_2_S*_x_*, 2 ≤ *x* ≤ 4) and is ultimately believed to produce insoluble Li_2_S_2_/Li_2_S. Once in the electrolyte, PS intermediates are free to diffuse to the Li anode or deposit on the cathode surface. During charging, high order PS intermediates may diffuse to the anode and react with lithium to produce lower order PS species. The soluble low order PS may then diffuses back to the cathode generating high order PS. This cyclical process that interconverts high to low order PS in the electrolyte is termed shuttling and consumes the active material without generating electrical energy. It leads to Li–S cells with unacceptably low coulombic efficiency.^15^, ^16^ Additionally, during discharge, the redox product Li_2_S, which is both electronically and ionically insulating, can deposit on the cathode.^9^ Consequently, once a thin layer of Li_2_S is formed, lithiation is effectively suppressed resulting in a rapid voltage decline, prematurely ending the discharge.

To prevent sulfur dissolution, many researchers have modified the cathode to sequester Li_2_S*_x_* using interactions with carbon and other species. Stable cycling performance has also been observed through the incorporation of mesoporous silica in the cathode.^13^ An encapsulating carbon matrix is has been broadly studied and is thought to trap PS intermediates within the porous matrix, limiting PS shuttling. The effectiveness of the silica trapping mechanism has led some researchers to propose engineered cathodes using both mesoporous metal oxides and carbon.^17^, ^18^, ^19^ Novel cathode modification strategies based on binder selection have also been reported.^7^, ^20^ Cui and co‐workers showed that a polyvinyl pyrrolidone (PVP) binder in the cathode produces strong interactions between the nonpolar carbon and polar PS intermediates resulting in stable Li–S cell performance.^21^, ^22^ Subsequent calculations confirmed the binding affinity of PVP to be superior to more commonly used polyvinylidene fluoride (PVDF). This concept was recently extended to design Li‐S battery cathodes based on amine‐ and ionic‐liquid additives that were shown by Density Functional Theoretical (DFT) calculations to strongly bind PS intermediates.^23^ Such additives have been reported to improve sulfur utilization and reduced capacity fade, but typically result in capacities about half (800 mA h g^−1^) of the theoretical value for Li‐S cells.

Electrolyte formulations that reduce PS dissolution are also a commonly practiced approach.^24^ High solvation electrolytes, such as tetraglyme, improve sulfur utilization with high initial discharge capacities (1000 mA h g^−1^), but low capacity retention.^25^, ^26^ Incorporation of PS into the electrolyte is another practiced approach to reduce sulfur loss from the cathode for improved utilization. In a complete series of studies, Zhang and co‐workers demonstrated that Li_2_S_9_ as a catholyte in Li–S cells yields improved cycling at moderate capacities (≈600 mA h g^−1^).^27^, ^28^, ^29^ Initial observations of PS shuttling were resolved with the addition of LiNO_3_ as a co‐salt with ability to protect the anode and prevent shuttling.^27^ High performance catholytes using low Li_2_S_5_ loadings were demonstrated in the work of Tarascon and co‐workers.^30^ Subsequent studies have incorporated similar catholytes designs in flow batteries,^31^ as salt additives,^32^ as well as in conventional Li–S cells.^33^

Since dissolved polysulfide must travel through the electrolyte and separator, the interactions of Li_2_S*_x_* with the separator might be expected to play an important role. Only recently have there been studies demonstrating the effective use of conductive‐coatings,^34^ single‐ion conducting nafion‐coatings,^35^ and layer‐by‐layer deposited polymer coatings^36^ on the polypropylene separator to increase sulfur utilization. These works show that the widely used polypropylene separator in its current state leads to capacity fade in Li–S cells. Similarly, interlayers have been explored to improve Li–S performance. A bifunctional carbon interlayer has shown increased performance as a localized PS trap to increase capacity retention.^37^ The placement of a graphene‐coated stainless steel mesh on the Li anode has led to similarly significant performance improvements.^38^ The improved performance of interlayers that localize or prevent the passage of PS underscores the need for a deeper focus on the separator design in Li–S cells.

Herein, we report on a Li–S cell model that allows the role of the separator in the cycling behavior of the battery to be isolated and studied. First, we consider a traditional Li–S cell with S_8_ sequestered in the cathode and find that when the cell is operated in a membrane‐less mode using an O‐ring style PTFE (polytetrafluoroethylene) separator, as much as a 40% improvement in discharge capacity is obtained with respect to cells that utilize a standard polypropylene, Celgard, membrane separator. In search of physical understanding, we study cells in which all of the sulfur in the form of Li_2_S_5_ is initially dissolved in the electrolyte. Again we find that the membrane‐less cell demonstrates improved capacity and cycling behavior. We show that these benefits may be preserved in Li–S cells that employ polypropylene separators with larger pores than those in current widespread use, showing for the first time that there are clear benefits for tailoring the separator pore size for optimized functioning of Li–S cells.

Hierarchical, silica etched carbon (SEC) particles were used as the cathode substrate in our studies.^39^ The selection of SEC particles is motivated by the affinity between PS intermediates and carbon, as well as any silica‐based residues left behind by incomplete etching. Characteristic N_2_ absorption and desorption isotherms for the as‐prepared materials are shown in **Figure** **1**a. A Brunauer–Emmett–Teller (BET) specific surface area of 921 m^2^ g^−1^ and a narrow pore size distribution centered at around 12 nm, Figure 1b, are observed. TEM analysis of the carbon matrix before (Figure 1c) and after etching (Figure 1d) shows that the NaOH etch produces a uniform distribution of pores, consistent with the BET data.

**Figure 1:**
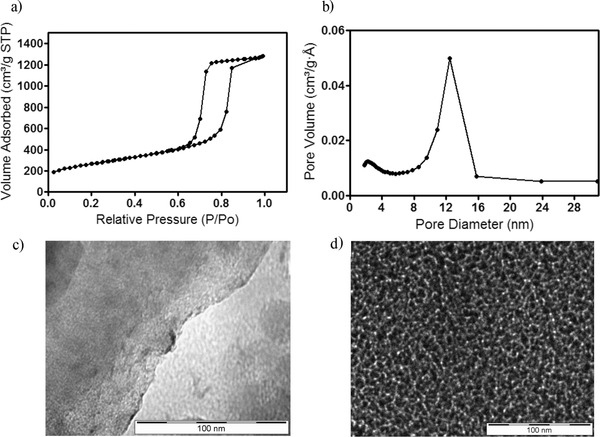
Surface area and morphology of silica etched carbon (SEC). a) Pore size distribution of SEC carbon, b) adsorption/desorption isotherm, and c) transmission electron microscope image of SEC particles before and d) after etching.

Chemical reduction of sulfur (S_8_) by metallic lithium to produce lithium polysulfides (Li_2_S*_x_*) has been demonstrated in so‐called catholyte‐based Li–S cells for over 30 years.^40^, ^41^ In this work, sulfur was treated with stoichiometric amounts of Li and Li_2_S, in the presence of tetraglyme, to yield Li_2_S_5_. These cells were studied in both a traditional membrane‐based format (**Figure** **2**a), using a commercial polypropylene (Celgard) separator, and in a membrane‐less configuration (Figure 2b), using a PTFE O‐ring spacer as the separator. In both the membrane‐based and membrane‐less cells, the lithium anode was presoaked with 10 wt% LiNO_3_ (in tetraglyme) for 24 h to passivate the anode prior to contacting it with the electrolyte containing Li_2_S_5_. Additionally, for the membrane‐based cells, the Celgard separator was presoaked with 10 wt% LiNO_3_ (in tetraglyme). Since Li‐PS is known to facilitate polysulfide shuttling, this is a critical step in preparing catholyte‐based Li–S cells as the Li‐PS in the electrolyte makes direct contact with the lithium metal. The spacer is pressed directly onto the lithium electrode creating a 28 μL lithium‐PTFE pouch in which the liquid catholyte is contained. In both cases 28 μL of electrolyte (≈0.7 mg Li_2_S_5_) was used as the active material to make our catholyte Li–S battery. As such, the ratio of Li_2_S_5_ to SEC used in a typical cell is equivalent to a sulfur loading of approximately 40 wt% based on the overall cathode mass.

**Figure 2:**
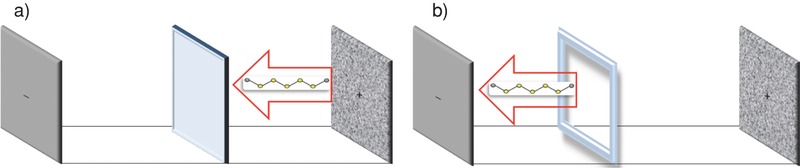
Schematic Li–S battery design with modified separator. a) Illustration of system with active material placed on top of the membrane on the cathode side. b) Illustration of catholyte system without membrane and active material placed inside the anode‐PTFE pouch.

Previously we reported that integration of nitrile‐containing additives in the sulfur cathode of a Li–S cell limits Li‐PS loss to the electrolyte via strong interactions between Li^+^ and the lone pair of electrons on nitrogen containing species.^23^ **Figure** **3** compares the galvanostatic cycling performance and discharge profiles at a fixed rate of C/5 of conventional Li–S cells in which the cathode is a physical mixture of 50 wt% sulfur, 40% carbon, and 10% binder and a conventional polypropylene material as separator. The baseline cell (green symbols) uses PVDF as binder and, as expected, displays a modest discharge capacity that fades quickly with cycling. In contrast, cells in which a 50/50 blend of the nitrile‐rich polymer, polyethylenimine (PEI), and PVDF is used as the binder for the cathode display substantially higher capacities, but exhibit similar capacity fading overtime, implying that while the PEI additive improves sulfur utilization in the cathode, access to the sulfur becomes progressively worse over multiple cycles of charge and discharge. The red symbols are the corresponding results for a Li–S cell in which the PEI/PVDF blend is used as binder, but which uses a PTFE O‐ring in our membrane‐less design. The capacity in the first cycle is similar in both membrane‐less and with membrane systems. However, with increasing cycle number, capacity fading is visibly less pronounced in membrane‐less cells, achieving a capacity of over 800 mA h g^−1^ by the 100th cycle. Observation of the electrolyte shows that in all three cases at the 1st cycle, the electrolyte is colorless, but after the 1st cycle, the electrolyte becomes visibly colored in all cases indicating certain amount of sulfur species is dissolved into the electrolyte. During the first few cycles, cells with the propylene membrane separator deliver higher discharge capacities than those without a separator; however, with increased cycling, the discharge capacity of the membrane‐less system first eclipses then surpasses that of cells in which a membrane is used. This behavior is clearly indicated by the much weaker slope of the capacity versus cycle number plot for the membrane‐less cells.

**Figure 3:**
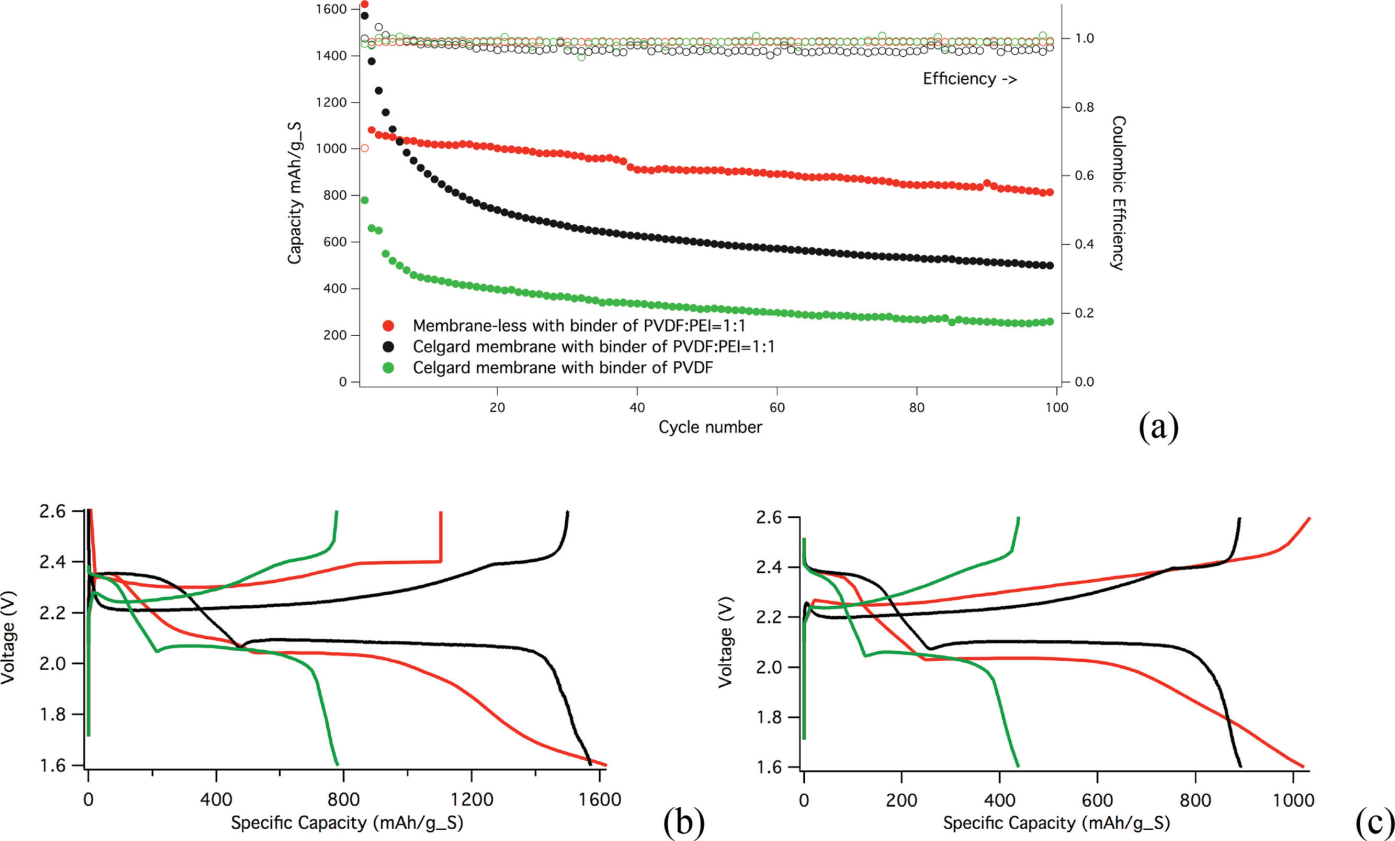
Electrochemical characterization of conventional Li–S cell. a) The cycling performance and coulombic efficiency of the membrane‐based (black and green) versus membrane‐less (red) cells at C/5 (the first conditioning cycle was run at a lower rate of C/50 and then ramped up to C/5), b) 1st cycle of the membrane versus membrane‐less system at C/50, and c) 10th cycle voltage profile at C/5.

These observations can be understood in terms of a competition between passivation of the lithium metal surface by dissolved Li‐PS and entrapment and transport limitations of dissolved Li‐PS in the polypropylene membrane. Specifically, we expect that the free passage of Li‐PS in the interelectrode space leads to faster reaction with the lithium metal to form an insulating Li_2_S*_x_* layer on the surface, which increases the interfacial impedance and can cause the initially lower capacity for the membrane‐less system. However, as cycling progresses, the concentration of the dissolved Li‐PS increases and this passivation effect will affect performance of all cells—whether or not they use a separator membrane. At the same time, the increased concentration of Li‐PS in the electrolyte may lead to a higher viscosity of the electrolyte, particularly in the boundary layer with the separator where free diffusion of Li‐PS is no longer possible. This will lead to Li‐PS hold‐up in the separator and a progressively lower ion mobility, which would cause both the sulfur utilization and capacity to fall in cells that utilize a separator membrane.

These ideas are supported by comparison of the voltage profiles for the membrane‐based and membrane‐less cell configurations. For the membrane‐based cells, there is a small dip at the beginning of the 2nd plateau, indicating the presence of Li‐PS precipitates in solution that results in the highest viscosity as a combined result of the S‐S chain length and number (concentration) of PS anions in solution.^29^ The sharp slope at the end of the discharge process is traditionally thought to reflect the insulating layer formed at the electrodes, leading to a faster drop of the voltage at fixed current. In comparison, the membrane‐less cell does not show the first dip in voltage, implying that the resistance increase due to an enhancement of viscosity is likely localized at in the separator pores near the electrolyte/separator interface. The disappearance of the dip is also seen to be accompanied by a more gentle slope at the end of the discharge, again indicating that the passivation effect traditionally associated with the electrodes is also a consequence of Li‐PS crowding and hold‐up in the separator membrane.

To evaluate these effects further, we investigated interfacial behavior and galvanostatic cycling features of membrane‐based and membrane‐less Li–S cells in which all of the active sulfur species (Li‐PS) are dissolved in the electrolyte. In the membrane‐based cell, Figure 2a, 28 μL of Li_2_S_5_ containing electrolyte was dropped onto the cathode before insertion of the membrane. Similarly, in the membrane‐less cells, 28 μL of Li_2_S_5_ containing electrolyte was dropped directly onto the anode. Electrochemical impedance spectroscopy (EIS) was performed on composite Li‐PS cells to investigate their interfacial properties when operated with/without a separator membrane. The impendence was also monitored every 24 h at OCV without cycling. **Figure** **4**a,b reports the transformation of the EIS spectra over time for the membrane‐based and membrane‐less systems. They both produced a characteristic semicircle associated with surface layer formation and evolution on the lithium anode. The start of the semicircle corresponds to the bulk resistance of the electrolyte and the width of the semicircle is proportional to the resistance of the surface layer on the electrode. The width of both semicircles, more clearly shown by the inset, is initially ≈200 ohm cm^2^ for both systems (Celgard:150 ohm cm^2^, PTFE O‐ring spacer: 200 ohm cm^2^). In the membrane‐based cell, Figure 4a, the semicircle maintains a stable width upon aging. The stable width of the impedance spectra with time indirectly indicates the resistance on the surface layer is stable due to the lack of interfacial chemical reactions between the PS intermediates in the electrolyte and the LiNO_3_ treated Li metal. Since PS intermediates are known to react with the Li surface, the absence of any increase in the interface resistance suggests PS intermediates at OCV diffuse very slowly, at best, through the separator membrane. In contrast, the ability of PS intermediates to form a resistive surface layer on the lithium electrode is clearly demonstrated by the increased width of the semicircle in the EIS spectra of membrane‐less cell (Figure 4b). Since the start of the semicircle relates to the bulk resistance of the electrolyte, this data is extracted and presented in **Table** [[qv: **1**]] where it is divided by the thickness of the two spacers to obtain bulk resistivity. Table 1 clearly illustrates that the bulk resistivity is much greater for the membrane cell.

**Table 1:** Bulk resistivity

	Bulk impedance [ohm cm^2^]	Thickness [cm]	Bulk resistivity [ohm cm]
Celgard member	18	0.0025	7200
PTEE Spacer	90	0.076	1184

**Figure 4:**
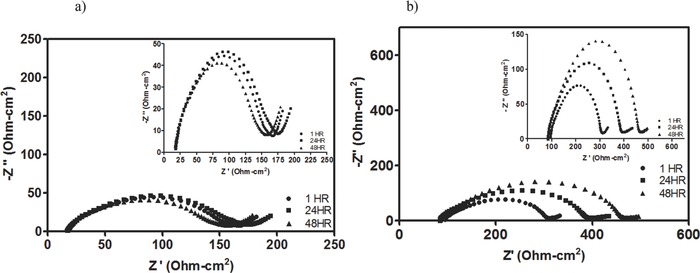
Electrochemical characterization of the Li/PS cell. a) The impedance spectroscopy of the polypropylene membrane at rest for 3 days. Inset: Magnification of EIS data; b) impedance spectroscopy of membrane‐less system at rest for 3 days. Inset: Magnification of EIS data.

Galvanostatic discharge/charge profiles of Li‐PS cells are reported in **Figure** **5**a–d. Since Li_2_S_5_ is used as active material, the relevant redox reaction is 8Li + 1Li_2_S_5_ → 5Li_2_S. The theoretical capacity is therefore 1340 mA h g^−1^ of sulfur, i.e. about 80% of a pure sulfur cathode. In the first charge, all cells are charged to 80% of the theoretical hours as an initialization step with subsequent cycles charged to a cutoff voltage of 2.53 V. Conventional wisdom states that cells discharged below 1.75 V in the presence of LiNO_3_ undergo substantial irreversible capacity loss. The membrane‐based cell under such conditions is limited to a capacity of 400 mA h g^−1^ after 50 cycles when discharged to 1.6 V (Figure S1, Supporting Information). Here we contest this wisdom and challenge the membrane‐less cell to harsher conditions by discharging to 1.6 V. The initial voltage profile for the membrane‐based Li‐PS cell, Figure 5a, exhibits all of the characteristics associated with Li–S, including the aforementioned sharp dip at 2.0 V, followed by a plateau region attributed to the formation of low order PS, and a decline in voltage below 2.0 V associated with the formation of insoluble Li_2_S_2_/Li_2_S. In contrast, the membrane‐less Li‐PS cell system maintains the characteristic shape, but the sharp dip around 2.0 V is again clearly missing. This behavior is similar to what is observed in membrane‐less cells where the sulfur is introduced as a solid composite in the cathode. Additionally, the extended plateau at ≈2.0 V is significantly more pronounced in the membrane‐less cell and the second initial plateau below 2.0 V is not present. As such, the membrane‐less system is able to discharge to significantly lower voltage without sacrificing performance. This is verified in the 50th voltage profile, Figure 5b, where the membrane‐less cell maintains its initial high capacity, whereas the membrane cell loses any capacity above the onset of the irreversible second plateau. Figure 5c reports the cycling behavior of both Li‐PS cell configurations at a fixed rate of C/5. The membrane‐less cells are again seen to outperform the membrane‐based Li‐PS cells in terms of stability and cyclability. The cycling performance of the membrane‐less cell at C/10 and C/5 is compared in Figure 5d.

**Figure 5:**
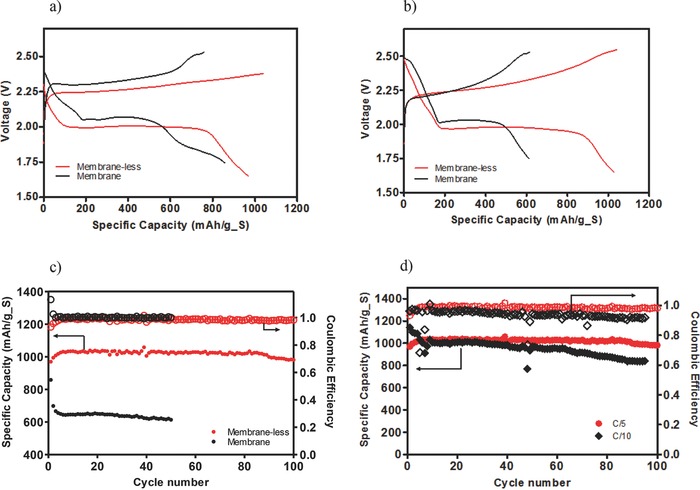
Galvanostatic discharge/charge cycling of the Li/PS cell. The voltage profile of the cells with Li_2_S_5_ for a) the 1st and b) the 50th cycle at C/5. c) The cycling performance and coulombic efficiency of the membrane versus membrane‐less systems at C/5. d) Cycling performance and coulombic efficiency of the membrane‐less cells at C/5 and C/10.

As with the Li–S cells discussed previously, we briefly studied the role of the binder affinity to PS intermediates on the performance of the membrane‐less Li‐PS cells. These studies are important because in the membrane‐less cell all contact between Li‐PS and the binder occurs via an electrolyte in which the Li‐PS is completely soluble. In particular, we compare a standard PVDF binder, which has limited affinity for Li‐PS, with a PVP binder that has been shown previously to interact strongly with LiPS species through binding of Li^+^. **Figure** **6**a shows that cells based on the PVP binder maintain a capacity of over 1000 mA h g^−1^ over 100 cycles of charge and discharge, compared with 600 mA h g^−1^ for the PVDF binder. Furthermore, we also probed the effect of cathode modification (PVP binder without LiNO_3_) versus anode protection (PVDF binder with LiNO_3_). The battery performance in Figure 6b shows that protection of the anode maintains high coulombic efficiency, but does not result in capacity retention. By modifying the cathode with a PVP binder, significantly higher capacity retention (800 mA h g^−1^) is observed although PS shuttling and low efficiency remains a problem. Thus, the binding affinity of PS intermediates with the cathode appears to have a greater effect on cycling performance compared with PS shuttling.

**Figure 6:**
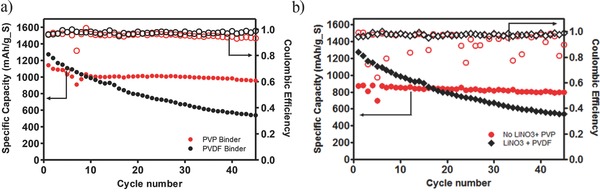
Effect of electrode and electrolyte additives on cycling behavior of membrane‐less Li‐PS batteries. a) Comparison of the effect of PVDF and PVP as binders in the cathode at C/10. b) Comparison of the effect of LiNO_3_ in the electrolyte versus PVP binder in the cathode at C/10.

Morphological changes at the lithium anode and SEC cathode following discharge of the Li‐PS membrane‐less cells were studied using SEM analysis. Although sulfur is initially charged to the electrolyte, **Figure** **7**a shows that a PVP bound carbon cathode is able to effectively capture sulfur compounds from the electrolyte as verified by EDX (Energy dispersive X‐ray spectroscopy) analysis shown in Figure 7b. Since each electrode is washed with 2 mL of tetraglyme after disassembly it is concluded that the sulfur species diffuse into the carbon matrix. SEM analysis of the lithium anode (Figure 7c) shows a clear buildup of insoluble precipitates on the surface in agreement with expectations from the large increase in interface resistance deduced from EIS analysis. The deposits on the Li metal anode are also seen to be limited to the circular open region of the PTFE O‐ring (Figure 7d) and the regions of the anode shadowed by the spacer are deposit free. Previous membrane‐based Li–S battery systems have shown a similar buildup of particulate material on the cathode surface after discharge.^9^, ^13^ However, by removing the separator, we find that the material exhibits a stronger affinity for the lithium anode and selectively deposits there. Surprisingly, the Li‐PS cell functions trouble free at a C/5 or C/10 rate under such conditions and the buildup of insoluble species on the Li anode does not appear to impact the performance of the cell at these rates. Considering the high interfacial resistance produced by the buildup of these species, however, it is expected that the effects of this buildup on cell performance will become worse at higher discharge/charge rates.

**Figure 7:**
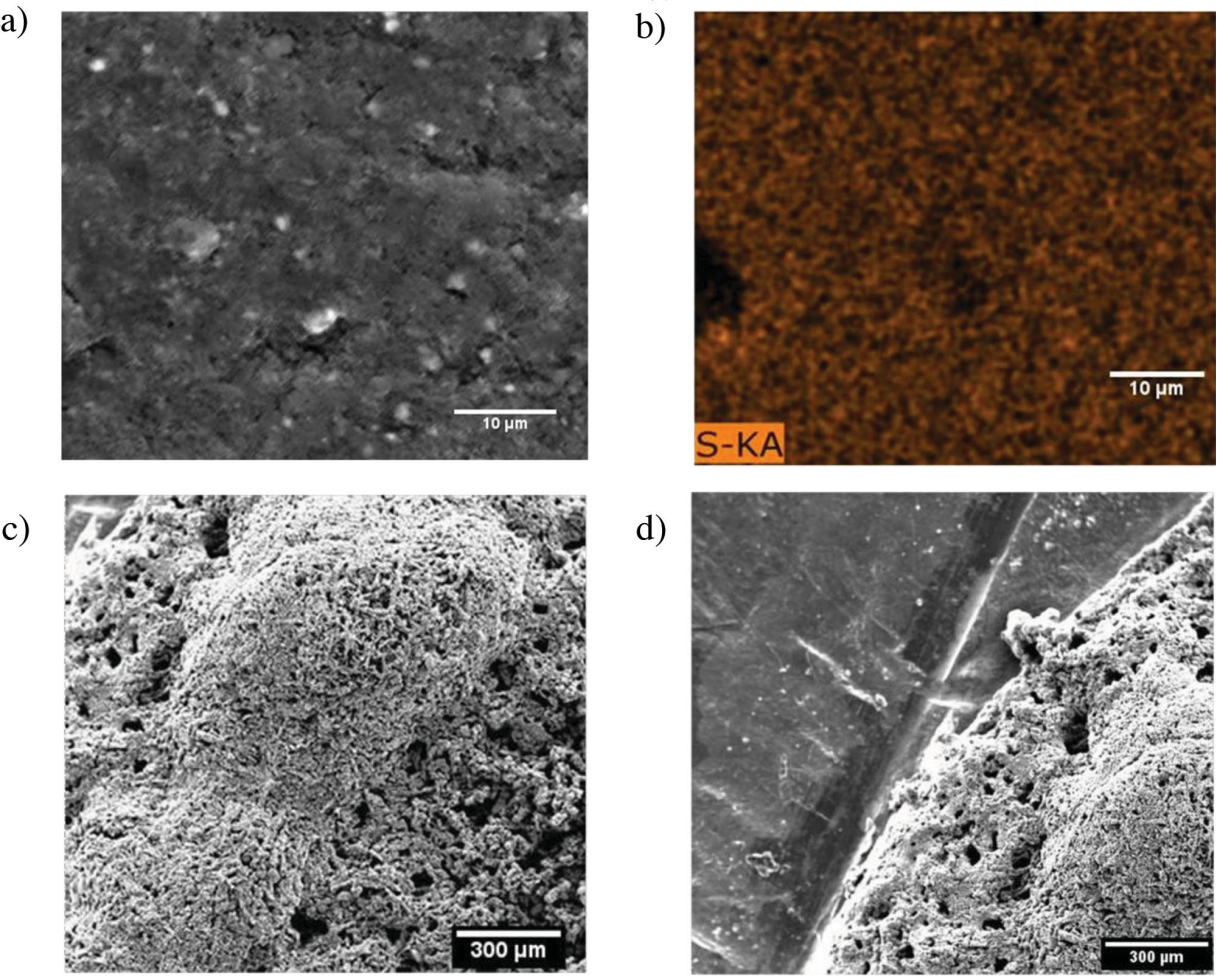
The morphology of the carbon and lithium electrodes. a) SEM image of the carbon cathode after 100 cycles. b) Elemental map of the sulfur EDX on the carbon surface after 100 cycles. c) SEM image of the lithium electrode after 100 cycles. d) SEM image of the lithium anode comparing the active surface and the inactive surface.

The chemical composition of the deposits seen in SEM analysis of the cathode was investigated using X‐ray photoelectron spectroscopy (XPS) analysis. The goal of XPS is to quantify the amount of polysulfide species, based on their relative intensities, which accumulate on the cathode for the membrane‐based and membrane‐less Li–S cell configurations. To begin the analysis, we studied the surface of the carbon cathode exposed to chemically synthesized high‐order Li‐PS species in the presence of LiTFSI. Next, XPS analyses were performed on the cathode surface after cells were cycled for 100 cycles. Each cathode was washed with 2 mL of tetraglyme and the characteristic features of the cathodes are shown in **Figure** **8**. All spectra contain the characteristic sulfur splitting of the S 2p signal into two components S 2p_3/2_ and S 2p_1/2_ separated by 1.12 eV with an intensity ratio of 2/1 as a result of spin‐orbital coupling effects. Based on the spectra obtained using cathodes soaked in electrolytes containing Li_2_S_9_ with LITFSI overnight (Figure 8a), we can attribute the peak formed at a binding energy of 169.5 eV to be LITFSI and the peak at 164.4 eV to S_8_ or Li_2_S_9_ species;^30^ the XPS peak for pure S_8_ is normally observed at 164 eV, and the XPS peaks associated with LiTFSI show a dominant S 2p_3/2_ peak at 169.5 eV. Previous XPS analysis of insoluble Li_2_S reveals a pair of peaks around a binding energy of 159 eV.^14^, ^30^, ^33^ Thus the species identified between 164 and 159 eV, at 162.7 (Figure 8b) and 162.9 eV (Figure 8c), for the membrane‐based and membrane‐less cells, respectively, are likely associated with insoluble Li‐PS species in the cathode. Additionally, it can be seen that the relative (relative to the LiTFSI peak, which can be used as a reference since both electrodes were pretreated in the same manner) intensities of XPS peaks associated with PS species are much higher in the case of the membrane‐less cells, which is consistent with the higher levels of sulfur utilization evident from the cycling studies and with the hypothesis that Li‐PS transport is limited by the separator membrane, which compromises Li–S battery performance.

**Figure 8:**
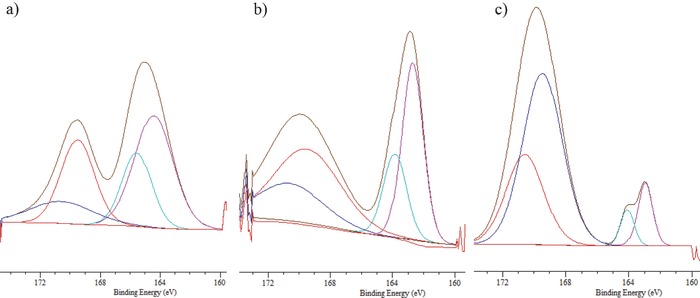
XPS spectra of cathode surface: a) Carbon cathode soaked in Li_2_S_9_ overnight. b) Carbon cathode used in membrane‐less Li‐PS cells after 100 cycles. c) Carbon cathode with membrane‐based Li‐PS cells after 100 cycles.

As a first step toward design of separators with optimized pore sizes for increasing performance of Li–S cells, we performed an initial study of membrane‐based cells containing separators with micron‐sized pores. **Figure** **9** illustrates one such example in which pores with an average diameter of ≈100 μm, from SEM analysis, are mechanically introduced in a standard separator. Preliminary analysis of the cycling performance (Figure 9c) and voltage profile (Figure 9d) shows features, including the absence of the initial fast capacity fade and lack of the sharp dip in the discharge voltage profile at the onset of the ≈2 V plateau, which are consistent with observations for the membrane‐less cells. A stable discharge capacity of ≈700 mA h g^−1^ is observed after 20 cycles with a coulombic efficiency ≈100%. We suspect that the lower capacity is a result of the low areal densities of the pores and will explore this in future studies. These early results nonetheless provide a path toward Li–S cells in which the pore characteristics of the separator are optimized to enhance transport of Li‐PS intermediates in the electrolyte and to improve sulfur utilization.

**Figure 9:**
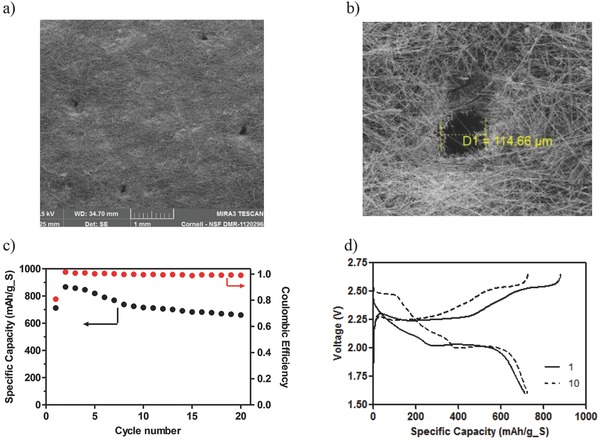
Characteristics of glass fiber membrane with large circular pores. a) SEM image of pore template in the separator. b) Average size of the manually created pore inside the separator. c) Cycling performance and coulombic efficiency at C/10. d) Voltage profile at C/10.

The separator in a Li–S rechargeable battery is shown to negatively affect the overall performance of the cell. Using both conventional Li–S cells in which sulfur is sequestered in the cathode as a carbon–sulfur composite and catholyte Li‐PS cells in which sulfur is introduced in the cell via soluble high‐order lithium polysulfides dissolved in the electrolyte, we show that it is possible to create membrane‐free Li–S cells with better sulfur utilization and more stable long‐term cycling features. We also find that in a membrane‐less cell design, the sharp drop in discharge profile near the beginning of the 2 V discharge plateau—traditionally associated with an increased electrolyte viscosity due to dissolution of high‐order Li‐PS species^29^—is either not observed or is significantly suppressed. This observation implies that the viscosity enhancement is likely associated with Li‐PS buildup at the electrolyte/separator interface or is a consequence of Li‐PS concentration in the small pores of the separator membrane. The membrane‐less cell is thought to significantly reduce diffusion barriers within the separator creating the easiest path of transport between the anode and cathode.

Once the membrane barrier is removed, stable capacity performance is shown to be maintained by integrating binder additives, such as PVP and PEI, which provide a mechanism for sequestering sulfur in the cathode. Finally, we find that insoluble PS intermediates that traditionally accumulate on the cathode and separator surface preferentially deposit on the lithium metal anode. Surprisingly, although these PS precipitates increase the interfacial resistance of the anode, they do not appear to affect cycling performance of the cell at a moderate discharge rate of C/5. As a first step toward a rationally engineered separator for Li–S cells, we show that Li–S cells containing a conventional separator modified to introduce large pores exhibit electrochemical features similar to our membrane‐less cells. The simplicity of our separator design concept is expected to lead to numerous follow‐on studies where synthesis approaches similar to those already in widespread use for engineering Li–S battery cathodes will be employed to produce optimized separators that facilitate LiPS transport and sulfur utilization in the Li–S cell.

## Experimental Section

*Materials*: Li_2_S_5_ catholyte was made by mixing predetermined amounts of sulfur (S_8_, 99.99%, Sigma‐Aldrich), Lithium (FMC, 99%, Sigma‐Aldrich), and Lithium sulfide (Li_2_S, 99%, Sigma‐Aldrich) in an Ar‐filled glove box. Sulfur was treated with stoichiometric amounts of Li and Li_2_S, in the presence of tetraglyme, to yield Li_2_S_5_. The Li‐PS mixture, Li_2_S_5_, was further diluted in the presence of tetraglyme to produce a 5 wt% solution of Li_2_S_5_. This solution was then combined with 1M LiTFSI in tetraglyme in a 1:1 weight ratio to produce electrolytes used for out Li‐PS studies. The ratio of Li_2_S_5_ to SEC carbon in the cathode constituted a sulfur loading of approximately 40 wt%. To synthesize the hierarchical SEC carbon material used for the cathode, commercial silica nanoparticles in water (LUDOX HS‐30 30 wt%, Sigma‐Aldrich) were combined with glucose in a 1:1 weight ratio and mixed for 5 h. The mixture was subsequently freeze‐dried and calcined at 1000 °C to produce a carbonized material. Silica etching was then performed in 5 m NaOH to produce SEC with a hierarchical pore structure. To create cathodes from this material, a slurry was created by blending SEC (80% by mass) and super P‐Li (10% by mass, SBET = 100 m^2^ g^−1^) together with polyvinylidene fluoride binder material (PVDF, 10% by mass, Sigma), PVDF/Polyethyleneimine (PEI), or polyvinylpyrrolidone binder material (PVP, 10% by mass, Sigma) dissolved in *N*‐methyl‐2‐pyrrolidone solvent. The obtained slurry was then coated on half inch aluminum foil disk and dried at 65 °C for 24 h. The typical sulfur loading amount was 1.1 mg.

*Electrochemical Analysis*: A two‐electrode CR2032 coin cell configuration was used for galvanostatic cycling. Traditional Li–S cells were assembled with Li foil (Alfa Aesar) as anode, sulfur–carbon composite as cathode (50% Sulfur, carbon, and 10 wt% polymer binder), and polypropylene, Celgard, membrane as separator. Catholyte cells were assembled with Li foil (Alfa Aesar) as anode, Li_2_S_5_ as catholyte, and SEC as cathode, in an Ar‐filled glove box. Polypropylene separators were soaked in 10 wt% LiNO_3_ (in tetraglyme). A Maccor Series 4000 battery tester was used for the battery tests. The capacity was normalized to the mass of sulfur. EIS tests were conducted using a Solartron CellTest System model 1470E potentiostat/galvanostat.

*Structure Characterization*: A LEO 1550 high‐resolution scanning electron microscopy was employed to characterize the morphology of the composite electrodes. XPS measurements were performed with a Surface Science SSX‐100 spectrometer using a monochromatic Al Kα source (1486.6 eV). Nonlinear least‐squares curve fitting was applied to high‐resolution spectra, using CasaXPS software.

## Supporting information

As a service to our authors and readers, this journal provides supporting information supplied by the authors. Such materials are peer reviewed and may be re‐organized for online delivery, but are not copy‐edited or typeset. Technical support issues arising from supporting information (other than missing files) should be addressed to the authors.

Supplementary

## References

[1] X. Ji , L. F. Nazar , J. Mater. Chem. 2010, 20, 9821.

[2] P. G. Bruce , S. A. Freunberger , L. J. Hardwick , J.‐M. Tarascon , Nat. Mater. 2012, 11, 19. 10.1038/nmat3191 22169914

[3] X. Ji , K. T. Lee , L. F. Nazar , Nat. Mater. 2009, 8, 500. 19448613 10.1038/nmat2460

[4] N. Jayaprakash , J. Shen , S. S. Moganty , A. Corona , L. A. Archer , Angew. Chem. 2011, 123, 6026. 10.1002/anie.201100637 21591036

[5] G. He , X. Ji , L. F. Nazar , Energy Environ. Sci. 2011, 4, 2878.

[6] Y. Yang , M. T. McDowell , A. Jackson , J. J. Cha , S. S. Hong , Y. Cui , Nano Lett. 2010, 10, 1486. 20184382 10.1021/nl100504q

[7] J. C. Guo , Z. C. Yang , S. Xu , Y. C. Yu , H. D. Abruna , L. A. Archer , J. Am. Chem. Soc. 2013, 135, 763. 23234561 10.1021/ja309435f

[8] S. W. Wei , W. Li , J. J. Cha , G. Zheng , Y. Yang , M. T. McDowell , P.‐C. Hsu , Nat. Commun. 2013, 4, 1331. 23299881 10.1038/ncomms2327

[9] Y. Yang , G. Zheng , Y. Cui , Chem. Soc. Rev. 2013, 42, 3018. 23325336 10.1039/c2cs35256g

[10] Y.‐X. Yin , S. Xin , Y.‐G. Guo , L.‐J. Wan , Angew. Chem. Int. Ed. Engl. 2013, 52, 13186. 24243546 10.1002/anie.201304762

[11] E. S. Shin , K. Kim , S. H. Oh , W. Il. Cho , Chem. Commun. 2013, 49, 2004. 10.1039/c2cc36986a 23223501

[12] G. He , S. Evers , X. Liang , M. Cuisinier , A. Garsuch , L. F. Nazar , ACS Nano 2013, 7, 10920. 24229005 10.1021/nn404439r

[13] X. Ji , S. Evers , R. Black , L. F. Nazar , Nat. Commun. 2011, 2, 325. 21610728 10.1038/ncomms1293

[14] Y.‐S. Su , Y. Fu , T. Cochell , A. Manthiram , Nat. Commun. 2013, 4, 2985. 24346483 10.1038/ncomms3985

[15] N. Jayaprakash , J. Shen , S. S. Moganty , A. Corona , L. A. Archer , Angew. Chem. 2011, 123, 6026. 10.1002/anie.201100637 21591036

[16] S. E. Cheon , K. K. Ko , J. H. Cho , S. W. Kim , E. Y. Chin , H. T. Kim ,J. Electrochem. Soc. 2003, 150, A796.

[17] Q. Pang , D. Kundu , M. Cuisinier , L. F. Nazar , Nat. Commun. 2014, 5, 4759. 25154399 10.1038/ncomms5759

[18] K. T. Lee , R. Black , T. Yim , X. Ji , L. F. Nazar , Adv. Energy Mater. 2012, 2, 1490.

[19] M.‐K. Song , Y. Zhang , E. J. Cairns , Nano Lett. 2013, 13, 5891. 24219588 10.1021/nl402793z

[20] M. J. Lacey , F. Jeschull , K. Edström , D. Brandell , Chem. Commun. 2013, 49, 8531. 10.1039/c3cc44772c 23942571

[21] G. Zheng , Q. Zhang , J. J. Cha , Y. Yang , W. Li , Z. W. She , Y. Cui , Nano Lett. 2013, 13, 1265. 23394300 10.1021/nl304795g

[22] Z. W. Seh , Q. Zhang , W. Li , G. Zheng , H. Yao , Y. Cui , Chem. Sci. 2013, 4, 3673.

[23] L. Ma , H. Zhuang , Y. Lu , S. S. Moganty , R. G. Hennig , L. A. Archer , Adv. Energy Mater. 2014,14, 1400390.

[24] J. Scheers , S. Fantini , P. Johansson , J. Power Sources 2014, 255, 204.

[25] C. Barchasz , J.‐C. Leprêtre , S. Patoux , F. Alloin , Electrochim. Acta 2013, 89, 737.

[26] C. Barchasz , J.‐C. Leprêtre , S. Patoux , F. Alloin , J. Electrochem. Soc. 2013, 160, A430.

[27] S. S. Zhang , J. A. Read , J. Power Sources 2012, 200, 77.

[28] S. S. Zhang , D. T. Tran , J. Power Sources 2012, 211, 169.

[29] S. S. Zhang , J. Power Sources 2013, 231, 153. 10.1016/j.jpowsour.2013.01.012 PMC4389686 25866437

[30] R. Demir‐Cakan , M. Morcrette , A. Guéguen , R. Dedryvère , J.‐M. Tarascon , Energy Environ. Sci. 2013, 6, 176.

[31] Y. Yang , G. Zheng , Y. Cui , Energy Environ. Sci. 2013, 6, 1552.

[32] R. Xu , I. Belharouak , J. C. M. Li , X. Zhang , I. Bloom , J. Bareño , Adv. Energy Mater. 2013, 3, 833.

[33] C. Zu , Y. Fu , A. Manthiram , J. Mater. Chem. A 2013, 1, 10362.

[34] H. Yao , K. Yan , W. Li , G. Zheng , D. Kong , Z. W. She , V. K. Narasimhan , Z. Liang , Y. Cui , Energy Environ. Sci. 2014, 7, 3381.

[35] J.‐Q. Huang , Q. Zhang , J.‐Q. Peng , X.‐Y. Liu , W‐Z Qian , F. Wei , Energy Environ. Sci. 2014, 7, 347.

[36] M. Gu , J. Lee , Y. Kim , J. S. Kim , B. Y. Jang , K. T. Lee , B. S. Kim , RSC Adv. 2014, 4, 46940.

[37] Y.‐S. Su , A. Manthiram , Nat. Commun. 2012, 3, 1166. 23132016 10.1038/ncomms2163

[38] C. Huang , J. Xiao , Y. Shao , J. Zheng , W. D. Bennett , D. Lu , L. V. Saraf , M. Engelhard , L. Ji , J. Zhang , X. Li , G. L. Graff , J. Liu , Nat. Commun. 2014, 5, 3015. 24402522 10.1038/ncomms4015

[39] L. Estevez , R. Dua , N. Bhandari , A. Ramanujapuram , P. Wang , E. P. Giannelis , Energy Environ. Sci. 2013, 6, 1785.

[40] R. D. Rauh , F. S. Shuker , J. M. Marston , S. B. Brummer , J. Inorg. Nucl. Chem. 1977, 39, 1761.

[41] R. D. Rauh , K. M. Abraham , G. F. Pearson , J. K. Surprenant , S. B. Brummer , J. Electrochem. Soc. 1979, 126, 523.

