# Supplementary material for: Model Membrane‐Free Li–S Batteries for Enhanced Performance and Cycle Life
Source: Adv Sci (Weinh). 2015 Apr 15;2(5):1500068. doi: 10.1002/advs.201500068 (PMC5115367; doi:10.1002/advs.201500068)
Supplement: Supplementary file 1 — Supplementary [file ADVS-2-0g-s001.pdf]

Copyright WILEY-VCH Verlag GmbH & Co. KGaA, 69469 Weinheim, Germany, 2015.

ADVANCED SCIENCE
Open Access

Supporting Information

for *Adv. Sci.*, DOI: 10.1002/advs.201500068

Model Membrane-Free Li–S Batteries for Enhanced Performance and Cycle Life

*Kenville E. Hendrickson, Lin Ma, Gil Cohn, Yingying Lu, and Lynden A. Archer**

Supporting Information

Model Membrane-free Li-S Batteries for Enhancing Performance and Cycle Life

*Kenville E. Hendrickson, Lin Ma, Gil Cohn, Yingying Lu, and Lynden A. Archer**

a) b)

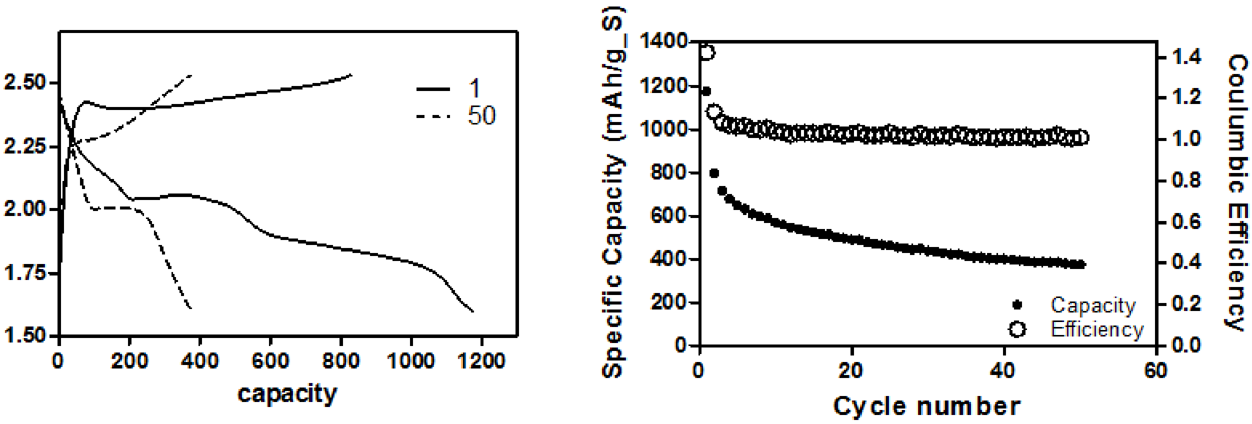

Figure S1: Membrane-based cell discharged to 1.6V. a) Voltage profile of the membrane-based cell with Li_2S_5 at C/5 for the 1st and 50th cycle. b) Cycling performance and coulombic efficiency at C/5.

a) b)

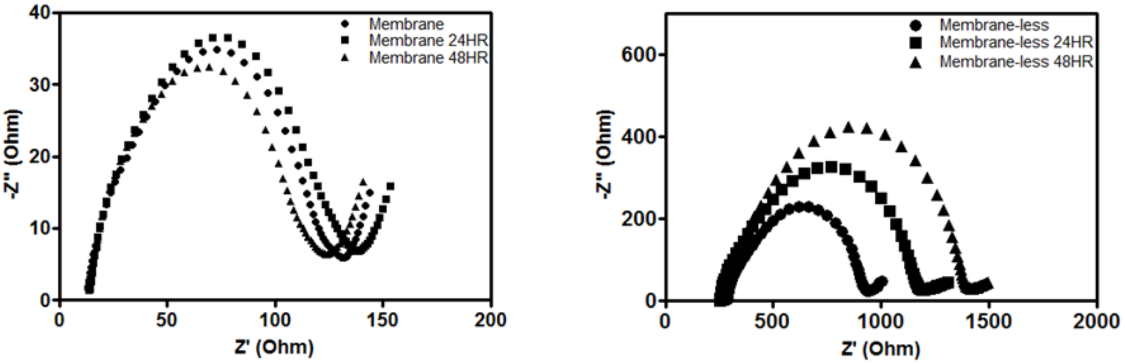

Figure S2: Electrochemical impedance spectroscopy raw data. a) cellgard membrane b) PTFE membrane-less spacer.
